# Fitting in, Standing out: Latent profiles of personal niche construction strategies at the workplace among Hungarian physicians

**DOI:** 10.1186/s12913-026-14317-4

**Published:** 2026-03-10

**Authors:** Orsolya Gyöngyösi, Tamás Simon, Tamás Martos, Viola Sallay

**Affiliations:** 1 Clinical Medical Sciences Doctoral School, Faculty of Medicine of the University of Szeged, Szeged, Hungary; 2 Unaffiliated, Szeged, Hungary; 3 Department of Personality, Clinical and Health Psychology, Faculty of Humanities and Social Sciences, University of Szeged, Szeged, Hungary; 4 Faculty of Psychotherapy Science, Sigmund Freud Private University, Vienna, Paris, France

**Keywords:** Physician, Healthcare environment, Workplace well-being, Personal niche construction, Latent profile analysis, Hungary

## Abstract

**Introduction:**

The present study examined the strategies of personal niche construction among Hungarian physicians at the workplace through basic psychological needs satisfaction and territoriality, within the socio-physical context of their workplace. The study aimed to delineate the latent patterns underlying participants’ organization, to characterize the attributes differentiating each subgroup, and to examine how these distinctive configurations contributed to strategies of niche construction within the workplace.

**Methods:**

A cross-sectional online survey was conducted among Hungarian physicians who have at least a degree in medicine and are currently pursuing medical practice (*N* = 261). Participants completed validated instruments assessing basic psychological needs satisfaction, workplace territoriality, and occupational well-being. Exploratory factor analysis identified underlying dimensions of need fulfillment and territoriality, followed by latent profile analysis to classify physicians into subgroups based on these patterns. Multinomial logistic regression and ANOVAs assessed demographic predictors and group differences in work satisfaction, fulfillment, interpersonal disengagement, exhaustion, and turnover intention.

**Results:**

A three-profile solution emerged, reflecting distinct personal niche construction strategies: (1) *Effortful self-expression* (2), “*Effortless self-expression”*, and (3) “*Low engagement in self-expression”*. The profiles were named based on the quality of person-environment fit. Profiles differed significantly in basic psychological needs satisfaction, professional fulfillment, and interpersonal disengagement (p < 0.01). Profiles also differed significantly in work satisfaction, professional fulfillment, and interpersonal disengagement, with the “Low engagement in self-expression” group reporting the least favorable outcomes.

**Conclusion:**

The findings highlight the relevance of socio-ecological perspectives in occupational health, demonstrating that physicians’ capacity to construct, claim, and maintain their professional niches serves as a central mechanism of resilience in complex healthcare systems. Supporting autonomy, competence, and balanced ownership of workspace may sustainably foster physicians’ well-being and engagement.

**Supplementary Information:**

The online version contains supplementary material available at 10.1186/s12913-026-14317-4.

## Introduction

### Create your own niche – even at your workplace

**Fig. 1:**
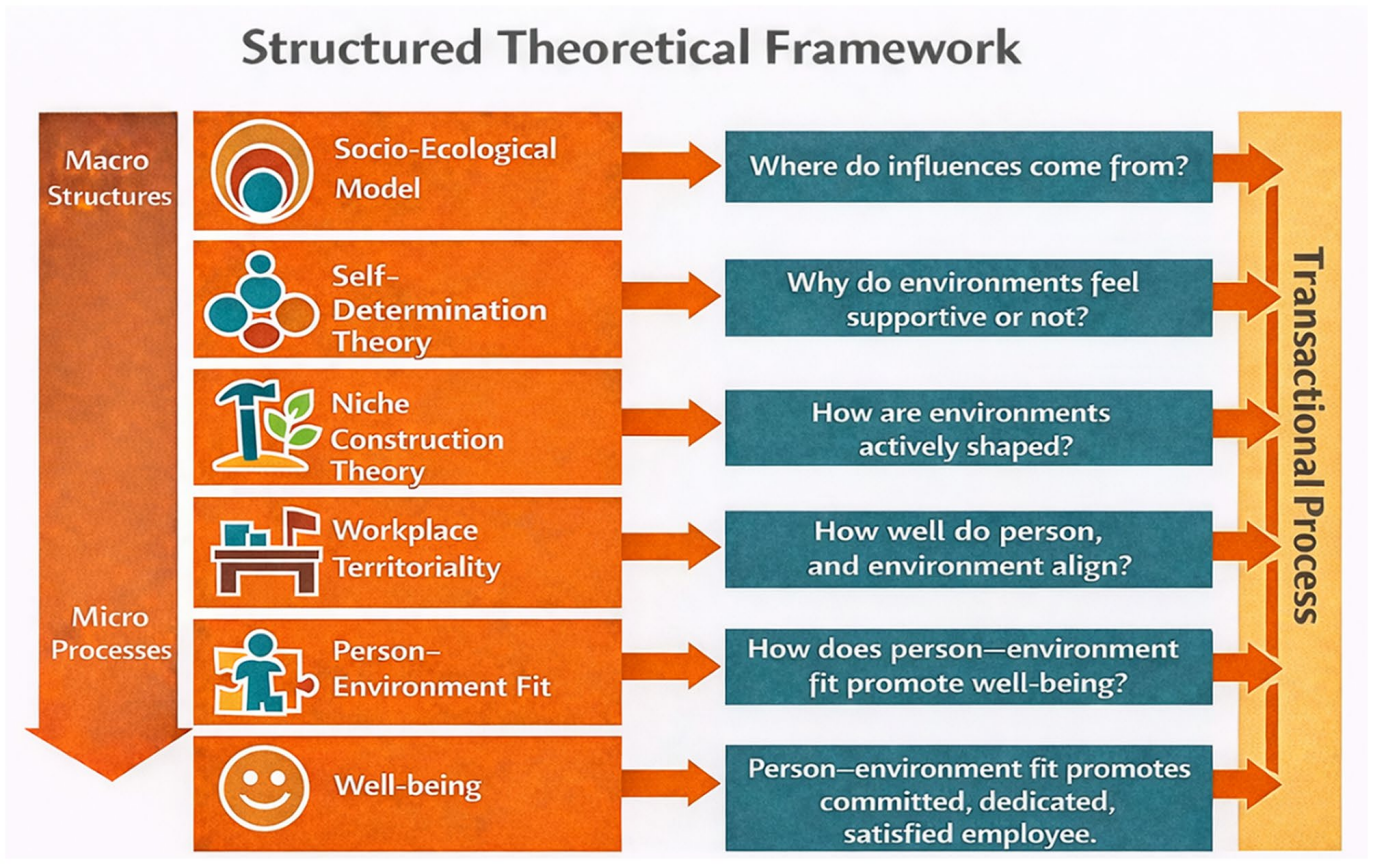
The multi-layered embeddedness of workplace well-being

How can physicians create their personal niches at work, and why is this of importance for their well-being? Recent research increasingly highlights physician well-being as a decisive factor not only for physicians’ health, but also for workplace climate, professional performance, and physicians–patient relationships [1]. International trends are shifting from focusing solely on problems and pathomechanisms (e.g. occupational distress and burnout research) toward identifying resources and strategies that help physicians remain healthy and balanced [2]. The COVID-19 pandemic further reinforced the importance of well-being as a foundation for physicians’ capacity to serve patients and for the mission of healthcare organizations [3]. Physician well-being is a complex and contested concept. Definitions typically encompass emotional health, physical health, a sense of challenged, spiritual well-being, work-life balance, thriving, authentic expressiveness and vigor [4]. Its negative counterpart—unwellness—refers to conditions such as emotional exhaustion, fatigue, anxiety, and depression, which undermine concentration and increase the risk of burnout. Our research assumed that one way to achieve well-being is through niche construction [5]. Rooted in positive psychology, this theory suggests that well-being depends on how individuals can create personal niches suited to their needs and qualities in continuous interaction with their environment [6]. We approached this through two perspectives: first, by examining workplace well-being via a socio-ecological lens; and second, by conducting a person-oriented analysis among Hungarian physicians to explore differences in how they create their personal niches. In the following, we use the systemic perspective of socio-ecological model to present how workplace well-being is embedded in various structures. See Fig. 1 for a graphical representation.

### The dynamic relationship between the Self and the environment

The *social-ecological model* builds on interaction between individuals and environments. Rooted in Bronfenbrenner’s ecological systems theory [7], it frames well-being as an outcome of processes spanning multiple levels, from the individual to the societal [8]. The model emphasizes that proximal processes—regular, reciprocal interactions between individuals and their environments—are the primary engines of psychological development and well-being.

*Self-determination theory* (SDT) complements this framework by emphasizing that autonomy, competence, and relatedness are essential for optimal functioning [9]. Well-being at workplace is greatly influenced by the satisfaction of basic psychological needs [10]. This concept, which belongs to the metatheory of SDT, states that psychological well-being and optimal functioning is predicated on autonomy, competence and relatedness [11]. Crucially, SDT focuses not on differences in needs across individuals, but on differences in how social environments meet those needs. Empirical studies confirm that satisfaction of these needs is linked to higher well-being and better functioning [5, 12]. Relational needs, grounded in attachment theory, further underscore the importance of supportive relationships as fundamental to well-being. In the workplace, social support fosters reciprocity, belonging, resilience, and stress management, thereby strengthening well-being [13]. In this sense, physical environments are not merely passive backdrops but serve as active mediums through which individuals construct, regulate, and communicate their identities, a dynamic process conceptualized as niche construction [14].

*Human niche construction* theory explores how people actively shape their social, physical, and technological surroundings [15]. A personal niche is a complex system in which spaces, relationships, and tools interact with the individual [6]. This active shaping can become central to job satisfaction and fulfillment [16].

Another viable way of implementing environmental self-regulation in the workplace is to manage the ownership of the space used by employees, i.e. *human territoriality*. Human territoriality, which also functions as a border regulation mechanism protects privacy, identity, and role communication, and is closely tied to psychological ownership—the sense that a space or object “belongs” to someone [17]. This provides control, efficiency, and familiarity [17]. Territoriality is inherently social, defined in relation to others, and plays a vital role in shaping workplace experience. In our research, we interpreted these needs as the relational aspect of personal niche construction, alongside spatial dimensions expressed through territoriality.

Building on this socio-ecological perspective, *person–environment fit* (P–E) theory provides a more fine-grained account of how individuals experience well-being within specific contexts. According to people-environment studies, the interaction between the individual and the physical environment fundamentally determines how we feel in a certain place [14]. The concept of P–E fit occupies a central position within people–environment studies [14]. A high degree of congruence between individuals and their environments has been shown to foster positive behavioral outcomes, attachment, and the development of place identity, processes that are further reinforced through sustained engagement with psychologically privileged places in our lives such as a workplace or home [18]. Emotional involvement and the consolidation of place identity have been found to support the fulfillment of basic psychological needs, particularly autonomy, competence, and relatedness. The satisfaction of these needs is sustained through mechanisms of environmental self-regulation, whereby individuals create and maintain personal spaces that both reflect and reinforce themselves [19, 20].

Conceptualizing the environment in socio-physical sense allows for a more nuanced understanding of how structural, material, and relational factors jointly shape individual experiences, and it provides a framework for identifying modifiable conditions that support sustainable and healthy professional functioning.

### Making space at work: environmental fit and territorial behavior

Certain types of environments play a prominent role in our lives and are significant from both an emotional and cognitive perspective—these are known as primary territories [14]. The P-E fit is environment-specific, so it is worth examining psychologically significant places based on the place-specific P-E fit that develops in them. Research shows that supportive environments buffer against burnout, while higher person–organization and person–group fit correlate with job satisfaction, commitment, and lower turnover or stress [21]. Job meaningfulness and turnover intention are central phenomenon to workplace well-being since they reflect employees’ evaluative attachment to their work and organization, integrating cognitive (psychological ownership), affective (relational closeness), and behavioral (territoriality) processes. When work is experienced as meaningful and “one’s own,” employees invest identity, energy, and relationships into the workplace, which strengthens commitment and reduces intentions to leave. Conversely, diminished meaning and weak relational or behavioral embedding undermine well-being by increasing psychological withdrawal and turnover intentions, making these constructs critical indicators of both individual and organizational health [21]. Studies on healthcare workers in China and internationally confirm that compatibility between individuals and their work environments enhances job embeddedness and satisfaction [22–24]. However, the literature has poorly explored this in Hungarian healthcare.

Workspaces strongly influence employees’ experiences, and many workers personalize or defend spaces to protect privacy and identity. Research identifies four types of workplace territorial behavior: personalization (e.g., placing family photos on a desk), control-oriented marking (e.g., leaving a coat on a chair), anticipatory defending (e.g., using passwords), and reactionary defending (responding to boundary violations) [25–27]. In healthcare settings, such behaviors support autonomy and competence, though defensive territoriality can hinder collaboration [28]. Shared territorial practices, by contrast, foster relatedness, and inclusion. In high-stress environments such as emergency rooms, balanced territorial control and supportive social ties can buffer burnout and enhance job satisfaction [29].

To complement these ecological perspectives, person-centered approaches can shed light on variations in personal niche construction strategies at the workplace among physicians. Latent profile analysis (LPA), a model-based method, for clustering individuals into unobserved groups. Person-centered approaches are relevant to physicians’ well-being research as they account for heterogeneity within the medical workforce, identifying distinct subgroups of physicians with differing well-being profiles and thereby informing more targeted and effective interventions. For instance, Zhu and colleagues identified three categories of physicians’ joy in work, with the majority reporting only medium or low levels. Those least happy showed unmet needs for autonomy, competence, and relationships, consistent with SDT’s predictions that unmet needs reduce engagement and raise burnout risk [30]. Similarly, Luna and colleagues distinguished four burnout-engagement profiles among health professionals, finding that multiple jobs, irregular shifts, and marital separation increased vulnerability to burnout, while factors such as job satisfaction, psychological well-being, and older age provided protection [31].

### Embedded in Europe: structural vulnerability of the Hungarian health workforce

Studying these phenomena in Hungary is particularly relevant because the national healthcare, social, and labor market contexts differ substantially from those of Western countries where the majority of relevant research has been conducted. Hungary faces long-standing challenges related to physician emigration, workforce shortages in rural regions, and career abandonment. It is therefore essential to examine not only the drivers of burnout and emigration, but also the factors that support remaining in the profession and in the country. Moreover, Hungarian research has traditionally emphasized deficit-oriented approaches, while protective factors, job satisfaction, work engagement, and professional fulfillment have received less attention. Therefore, context-sensitive research is needed to identify locally relevant predictors of well-being and sustainable medical careers [32].

### The current study

The present study examines basic psychological needs satisfaction (BPNs) and territoriality as elements of personal niche construction strategies of Hungarian physicians at the workplace and explores specific configurations of these factors using LPA. We aim to connect these distinct subgroups of physicians with their professional well-being, thereby capturing heterogeneity in personal niche construction strategies and their relation to work conditions.

## Methods

### Procedure

The current study was a cross-sectional survey; the sample consisted of physicians who have at least a degree in medicine and are currently pursuing medical practice. The recruitment period started on 11 October 2023, ended on 14 June 2024. We reached the participants by means of an online questionnaire. We created a questionnaire study using Lime Survey. Using purposive sampling, we sent a link of the questionnaire directly to individuals who met the criteria and asked them to share it further among medical professional groups (e.g., medical Facebook groups such as Hungarian Medical Chamber). All questions were obligatory; thus, we excluded partial answers.

### Ethical approval

The Ethical Board of the Hungarian Council of Health Sciences approved the study (nr. BM/15066- 3 /2023). We informed the participants in advance about the purpose of the study, explained that participation was voluntary, clarified that they could withdraw at any time, and stated that the data would be processed and published as aggregated statistical results. The investigation conforms to the principles outlined in the Declaration of Helsinki.

### Participants

A total of 689 participants opened the questionnaire, and 261 participants completed it. The sample included 175 females (68.6%), 79 males (31%) and one person did not want to give data about their gender (0.004%). The mean age of the participants was 45.4 years (SD = 12.7, min = 22, max = 82). We asked the participants about their current place of residence, their marital status, their level of qualifications, their work experience in healthcare, the type of healthcare institution in which they work and the current number of positions they hold as a medical professional (Table 1). Given that the inclusion criteria were having at least a degree in medicine and currently pursuing medical practice, we also asked about the type of specialty(ies). Table 2 gives the detailed distribution of the sample’s specialties.

**Table 1:** Sociodemographic and occupational characteristics of the participants

Sample characteristics	*N* (%)
**Place of residency**	
City with county rights	95 (36.4)
Capital	84 (32.2)
Town	56 (21.5)
Village/municipality	24 (9.2)
Other	2 (0.8
**Level of qualification**	
Intern	9 (3.4)
Resident	14 (5.4)
Specialist candidate	26 (10.0)
Specialist	138 (52.9)
Specialist with more than 1 specialty	74 (28.4)
**Marital status**	
Married	152 (58.2)
Partnership	46 (17.6)
Partnership without cohabitation	13 (5.0)
Dating w someone	5 (1.9)
Single	39 (14.9)
Do not know/do not want to answer	1 (0.4)
Other	5 (1.9)
**Years spent working in healthcare**	
0–5 years	34 (13.0)
6–10 years	36 (13.8)
11–15 years	46 (17.6)
16–20 years	23 (8.8)
More than 21 years	122 (46.7)
**Number of current positions**	
One	70 (53.0)
Two	40 (30.3)
Three	17 (12.9)
More than three	5 (3.8)
**Type of healthcare provider**	
Hospital	211
GP practice	128
Private healthcare provider	56
Specialist outpatient clinic	47
National Ambulance Service of Hungary	21
Outside of Hungary	19
Other	16

**Table 2:** Frequency of sample specialties

Type of specialty (multiple choice possible)	*N* (%)
Pediatrics	39 (13.3)
Family medicine	31 (10.6)
Internal medicine	30 (10.2)
Surgery	18 (6.1)
Orthopedics and traumatology	15 (5.1)
Psychiatry	14 (4.8)
Emergency and critical care medicine	13 (4.4)
Anesthesiology and intensive care medicine	12 (4.1)
Occupational medicine	12 (4.1)
Ophthalmology	11 (3.8)
Neurology	10 (3.4)
Psychotherapy	7 (2.4)
Cardiology	5 (1.7)
Pulmonology	5 (1.7)
Rheumatology	5 (1.7)
Clinical oncology	4 (1.4)
Infectious diseases	4 (1.4)
Obstetrics and gynecology	4 (1.4)
Endocrinology and metabolic diseases	4 (1.4)
School health and adolescent medicine	4 (1.4)
Child and adolescent psychiatry; Public health and preventive medicine; Oral and maxillofacial surgery; Dermatology; Vascular surgery; Gastroenterology; Hematology; Thoracic surgery; Military, disaster, and law enforcement medicine; Radiology; Pathology; Medical laboratory diagnostics; Addiction medicine; Health insurance medicine; Clinical pharmacology; Neonatology; Otorhinolaryngology; Geriatrics; Clinical genetics; Rehabilitation medicine; Medical microbiology; Urology; Allergy and clinical immunology; Andrology; Forensic psychiatry; Hand surgery; Clinical neurophysiology; Medical rehabilitation; Pediatric surgery; Nephrology; Forensic medicine; Neurosurgery; Plastic and burn surgery; Nuclear medicine; Transfusion medicine; Radiation oncology; Cardiac surgery; Audiology; Vascular medicine; Diagnostic cytology; Laboratory hematology and immunology; Molecular genetic diagnostics; Occupational hygiene; Neuroradiology; Gynecologic oncology (surgery); Sports medicine; Tropical medicine.	Each of these categories had n < = 3 (1.0%) respondents indicating them.

### Instruments

*Subjective rated health status - SRH*. We measured the respondent’s evaluation of their health status with the question “Overall, how would you rate your health?” The five response options ranged from very poor to excellent [33].

*Satisfaction with Work Scale – SWWS.* The Satisfaction with Work Scale (SWWS) was reworded from the Satisfaction with Life Scale (SWLS) [34, 35]. SWWS assesses the subjective experience of satisfaction with work. The questionnaire consists of 5 items rated on a 7-point Likert scale (1 = absolutely untrue, 7 = absolutely true). The questionnaire proved valid and reliable for both the original and the Hungarian versions [36]. Reliability indicators for the questionnaire used in the current study are Cronbach’s α = 0.86.

*Work and Meaning Inventory – WAMI.* The questionnaire was developed by Steger and colleagues [37]. The original version measures job meaningfulness through three dimensions: “Positive Meaning”, “Meaning Making through Work”, and “Greater Good Motivations”. The questionnaire consists of 10 items rated on a 5-point Likert scale (1 = absolutely untrue, 5 = absolutely true). The questionnaire proved valid and reliable for both the original and the Hungarian versions [38]. Reliability indicators for the questionnaire used in the current study are Cronbach’s α = 0.85, 0.76, 0.67, respectively.

*Basic Psychological Needs at Work Scale – BPNs.* The Basic Psychological Needs at Work Scale (BPNWS) is a revised version of the original English-language Need Satisfaction Scale (NSS), which was developed by Csordás and his colleagues [39]. The questionnaire assesses the satisfaction of the three basic psychological needs (autonomy, competence, and relatedness) formulated in Self-Determination Theory during work. A higher score on the scales indicates better satisfaction of needs. The questionnaire proved valid and reliable for both the original and the Hungarian versions [40]. Reliability indicators for the questionnaire used in the current study are Cronbach’s α = 0.77, 0.63, 0.80, respectively.

*Workplace Territorial Behavior Questionnaire - (WPTERR).* The original version of the Office Territorial Behavior Questionnaire was developed and published by Graham Brown [41]. In our study, we used the term “work area” (instead of workstation) which fits better into Hungarian healthcare nomenclature. The WPTERR contains four subscales, including (a) identity-oriented marking (six items, e.g., “I decorated the space the way I wanted to”), (b) control-oriented marking (five items; e.g., “I told others that this is my work area”), (c) anticipatory defending (six items; e.g., “I used locks and passwords to prevent others from accessing my work area”) and reactionary defending (six items; e.g., “I explained to the intruder that this work area was already occupied”). The questionnaire consists of 23 items rated on a 7-point Likert scale. The scale is scored by summing the responses to the items. Reliability indicators for the questionnaire used in the current study are Cronbach’s α = 0.85, 0.84, 0.84, 0.91, respectively.

*Perceived Office Privacy Questionnaire (PRIV.)* The questionnaire was originally developed by Oldham [42]. Oldham did not name the entire questionnaire in his original study; the six items in the questionnaire appear here on two scales. Laurence and colleagues [43] already use the instrument as a single scale, called Experience of privacy [1–6], and we did the same in our research. The authors of the Hungarian validation study named the questionnaire Perceived Office Privacy Questionnaire. The questionnaire consists of 6 items rated on a 5-point Likert scale. The scale is scored by summing the responses to the items. Items 3, 5 and 6 are reversed. The questionnaire proved valid and reliable for both the original and the Hungarian versions [44]. Reliability indicators for the questionnaire used in the current study are Cronbach’s α = 0.54.

*Stanford Professional Fulfillment Index (SPFI).* SPFI contains 16 + 4 items assessing experiences of professional fulfillment over the previous 2 weeks and it is divided into two main sections measuring the Professional Fulfillment Index and the Self-reported Medical Errors constructs. The Professional Fulfillment Index contains three subscales, including (a) professional fulfillment (six items; e.g., “I feel happy at work”), (b) job exhaustion (four items; e.g., “During the past two weeks I have felt a sense of dread when I think about work, I have to do”), and (c) interpersonal disengagement (six items; e.g., “During the past two weeks, my job has contributed to me feeling less empathetic with my patients”). The questionnaire does not contain reverse items and items are rated on a five-point Likert scale (0 = not at all, 4 = completely true). The scale is scored by summing the responses to the items, with a higher score indicating higher experience concerning the respective constructions. In the Self-reported Medical Errors section (four items), the respondents rated the recent occurrence of specific medical errors statements (e.g., “I made a major medical error that could have resulted in patient harm”) in their practice on a six-point Likert scale (0 = never, 5 = in the last week). The questionnaire proved valid and reliable for both the original and the Hungarian versions [1]. Reliability indicators for the questionnaire used in the current study are Cronbach’s α = 0.88, 0.85, 0.93, 0.65, respectively.

*Turnover intention* We measured the respondent’s intention of turnover with the question: “How often do you think about looking for a new workplace?” The questionnaire consists of one item rated on a 10-point Likert scale. (1 = never, 10 = very often).

### Data analysis

First, an exploratory factor analysis (EFA) was conducted on the combined items of the Basic Psychological Needs at Work Scale and the Workplace Territoriality Measure using maximum likelihood estimation with Oblique rotations method (Oblimin rotation), as correlations between factors were expected. The number of factors to retain was determined by a combination of parallel analysis, the scree plot, and the eigenvalue-greater-than-one criterion [45]. Factor loadings below |0.30| excluded for clarity [45]. Sampling adequacy was initially examined using the Kaiser-Meyer-Olkin (KMO) measure. The overall significance of the correlation matrix was assessed with Bartlett test and the factorability of the overall set of variables and individual variables, we used the measure of sampling adequacy (MSA) because it looks not only at the correlations, but also at patterns between variables [45]. Sampling adequacy was deemed acceptable with KMO values > 0.70, while Bartlett’s test of sphericity required significance (*p* < 0.001) [46]. Model evaluation followed established criteria, with RMSEA < 0.08 and TLI > 0.90 indicating acceptable fit [47].

Second, we performed latent profile analysis based on the standardized factor scores obtained from the preceding EFA. We compared multiple models that varied in the number of profiles and covariance structures. We selected the optimal solution—a three-profile model with unrestricted variances and covariances—based on fit indices Akaike Information Criterion (AIC), Bayesian Information Criterion (BIC), Bootstrap Likelihood Ratio Test (BLRT) entropy and interpretability. Lower values of AIC, BIC, indicate a better fit for the model. The entropy test values ranged from 0 to 1, with higher values indicating a better differentiation between profiles [48, 49].

Third, we subsequently conducted a multinomial logistic regression analysis to examine predictors of profile membership, entering gender, age, years of experience in healthcare, and professional status as covariates. Normality was assessed using the Shapiro–Wilk test. Although Shapiro–Wilk tests were statistically significant across outcomes, the magnitude of the test statistics indicated only mild deviations from normality for most variables (W values ranged from 0.947 to 0.986), which is expected in samples of this size due to the high sensitivity of the test. Homogeneity of variances was evaluated using Levene’s test. For two outcomes (professional fulfillment and exhaustion), Levene’s test indicated mild heterogeneity of variances (*p* = 0.041 and *p* = 0.017, respectively), whereas variance homogeneity was supported for the remaining outcomes. Given the robustness of ANOVA to moderate violations of these assumptions, particularly in large samples and in the absence of extreme group size imbalance, the analyses were considered appropriate. The smallest latent profile consisted of 27 participants, which was regarded as sufficient for reliable estimation of group means. In addition, according to the central limit theorem [50] sampling distributions of the means can be assumed to approximate normality even when raw score distributions deviate from normality. No data transformations or non-parametric alternatives were applied. Bonferroni-adjusted post hoc tests were used to ensure conservative inference.

Finally, differences between the identified profile groups were examined in relation to occupational well-being outcomes, such as work satisfaction, professional fulfillment, interpersonal disengagement, exhaustion, and intention to leave. We performed all analyses using JASP 0.19.3. (JASP Team, 2025).

## Results

### Exploratory factor analysis of scale items

We conducted a joint exploratory factor analysis on the thirty-two items of the Basic Psychological Needs at Work and Workplace Territoriality scales to identify the underlying dimensions of basic psychological needs satisfaction and territoriality. We systematically assessed the suitability of the data before conducting the exploratory factor analysis. Sampling adequacy was high (KMO = 0.888) indicating excellent suitability for factor analysis and Bartlett’s Test of Sphericity confirmed suitability (χ² = 4815, *df* = 496, *p* < 0.001). The MSA for all items exceeded 0.79, indicating that each item was suitable for inclusion in the factor analysis and contributed adequately to the overall factor structure [51]. Bartlett’s Test of Sphericity was significant (*χ²* = 4815, *df* = 496, *p* < 0.001), confirming that the correlation matrix was appropriate for factor extraction. The Basic Psychological Needs at Work Scale demonstrated good reliability (Cronbach’s α = 0.84), while the Workplace Territoriality Measure showed excellent reliability (Cronbach’s α = 0.90).

We performed factor extraction using maximum likelihood estimation with oblimin rotation to allow for correlated factors. The parallel analysis and the scree plot indicated a five-factor solution, as observed eigenvalues dropped below simulated data after the fifth factor. Although only the first four factors had eigenvalues greater than one (7.92, 4.67, 1.33, 1.07), the fifth factor (0.70) was retained based on these additional criteria. Model fit indices further supported this decision, with the five-factor solution demonstrating superior fit (RMSEA = 0.06, TLI = 0.872, BIC = -1200) compared to the three-factor model. The five-factor solution also provided a clearer and more interpretable structure, reflecting the original subscale structure of the Workplace Territoriality scale. The five retained factors together explained 53.7% of the total variance. Inter-factor correlations were low to moderate (ranging from 0.083 to 0.5), supporting the distinctiveness of the identified factors.

The factor loadings for each item are presented in Table 3. Items are grouped by their theoretical subscales. The analysis revealed that the first factor reflected basic psychological need fulfillment, as all items of the Basic Psychological Needs at Work Scale (BPNs) loaded strongly onto this factor. The remaining four factors corresponded to subdimensions of territoriality, with some overlap between subscales, particularly among control-oriented, anticipatory defending, and identity-oriented marking items.

Exploratory factor analysis revealed five distinct latent dimensions underlying basic psychological needs satisfaction and territoriality at work. **Factor 1**, *Basic Psychological Need Fulfillment*, comprises all Basic Psychological Needs at Work Scale items, reflecting overall satisfaction of autonomy, relatedness, and competence. **Factor 2**, *Identity-Oriented Marking* (with overlap), centers on personalization behaviors and includes elements of boundary creation and formal territorial recognition, indicating interconnections among identity reinforcement, spatial control, and ownership claims. **Factor 3**, *Control-Oriented Marking*, captures explicit, rule-based boundary marking practices. **Factor 4**, *Anticipatory Defending* (with overlap), primarily represents proactive defense strategies while encompassing communicative ownership behaviors, suggesting overlap between assertion and preemptive defense. **Factor 5**, *Reactionary Defending*, reflects defensive responses to territorial threats, characterized by hostility or avoidance.

In summary, the results of the factor analysis supported a five-factor solution characterized by clear and interpretable loadings, corresponding to theoretically meaningful constructs derived from both the Basic Psychological Needs at Work Scale and the Employee Territoriality Measure. We calculated factor scores using the regression method and then standardized them for the latent profile analyses.

**Table 3:** Factor loadings of joint exploratory factor analysis (BPNs and territoriality scales items)

			Factors				
Item	Subscale/ description	Item description	1	2	3	4	5
bpns_1	Autonomy	I feel free to decide what and how things happen in my work.	0.58				
bpns_2	Relatedness	I particularly like the people I come into contact with during my work.	0.60				
bpns_3	Competence	I feel that I am good at what I do.	0.43				
bpns_4	Autonomy	I feel that I can freely express my opinions and ideas at my workplace.	0.58				
bpns_5	Relatedness	I have a good personal relationship with those I regularly work with.	0.61				
bpns_6	Competence	Recently, I have managed to acquire interesting new skills.	0.49				
bpns_7	Competence	Most of the time, I feel fulfilled by what I do.	0.73				
bpns_8	Autonomy	I feel that I can often be myself in my work.	0.85				
bpns_9	Relatedness	My colleagues are generally quite friendly to me.	0.58				
wpter_1	Identity-oriented marking	Brought in personally meaningful photographs (e.g., friends, family, pets, etc.).		0.48			
wpter_2	Identity-oriented marking	Displayed artwork in my workspace.		0.75			
wpter_3	Identity-oriented marking	Brought in work related items (coffee mug, books).		0.67			
wpter_4	Identity-oriented marking	Decorated the space the way I wanted.		0.84			
wpter_5	Identity-oriented marking	Displayed things in the workspace that represent my personal hobbies/interests.		0.58			
wpter_6	Identity-oriented marking	Brought in items/changed workspace to make me feel at home.		0.76			
wpter_7	Control-oriented marking	Made a border around my workspace.		0.33	0.54		
wpter_8	Control-oriented marking	Told people about the boundaries of the workspace.			0.60		
wpter_9	Control-oriented marking	Made my name all over the workspace.			0.50		
wpter_10	Control-oriented marking	Used signs to communicate that the workspace is mine.			0.44	0.39	
wpter_11	Control-oriented marking	Told people the workspace is mine.			0.42	0.48	
wpter_12	Control-oriented marking	Delayed allowing others to use my workspace until it is clear to everyone.				0.77	
wpter_13	Control-oriented marking	Enlisted support of others to protect my space when not present.				0.87	
wpter_14	Anticipatory defending	Developed formal rules to protect workspace.				0.77	
wpter_15	Anticipatory defending	Avoid leaving workspace unattended.				0.68	
wpter_16	Anticipatory defending	Had authorities identify the workspace as mine.		0.36		0.37	
wpter_17	Reactionary defending	Used locks/passwords so others cannot access my workspace.				0.35	
wpter_18	Reactionary defending	Used facial expressions to express disagreement/dislike towards infringer.					0.74
wpter_19	Reactionary defending	Avoided working/interacting with infringer.					0.91
wpter_20	Reactionary defending	Explained to infringer that workspace was already claimed.					0.79
wpter_21	Reactionary defending	Devised a strategy to get back workspace from infringer.					0.77
wpter_22	Reactionary defending	Displayed hostility towards the infringer.					0.90
wpter_23	Reactionary defending	Complained to supervisor about the infringement.					0.60
Explained variance %			11.33	11.4	6.22	11.64	13.15
Factors score reliability			0.85	0.85	0.85	0.88	0.92

Note: only factor loadings above |0.30| are presented

### Latent profile analysis of psychological needs satisfaction and territoriality

The three-profile solution was characterized by high entropy (0.967), indicating clear separation between profiles. Additionally, the bootstrapped likelihood ratio test was significant (BLRT *p* < 0.01), supporting the presence of three distinct latent profiles. The model exhibited a strong fit to the data, as reflected by the Akaike Information Criterion (AIC = 2818.06) and the Bayesian Information Criterion (BIC = 3039.06), both indicating robust model adequacy. Collectively, these indices suggest that the selected model offers a robust and well-differentiated representation of the underlying patterns in the data. By allowing both variances and covariances to vary across profiles, the model afforded greater flexibility in representing the unique structural characteristics of each group. Detailed fit indices can be found in S1 in Supplementary Materials.

**Fig. 2:**
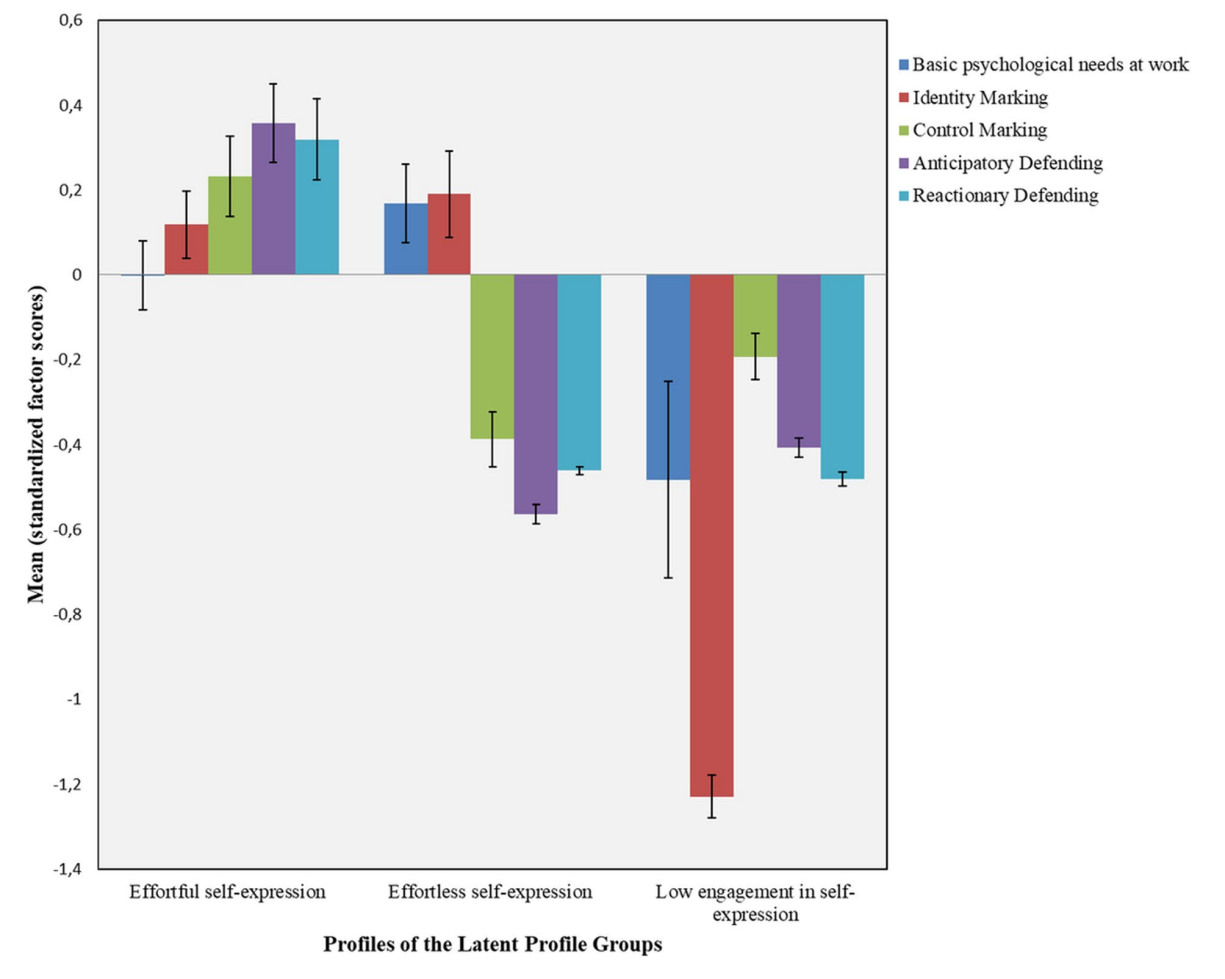
Profiles of the latent profile subgroups

The latent profiles identified in this analysis highlight diverse patterns of territorial behavior and basic psychological needs fulfillment among physicians. The distinct patterns of factor scores for each latent profile are illustrated in Fig. 1, which visually compares the standardized scores across the three groups. Profile 1 (“Effortful self-expression”) displayed moderate levels across all territoriality dimensions and moderate level of need satisfaction, suggesting that active assertion and defense of workspace is associated with a sense of autonomy, relatedness, and competence at work. In contrast, Profile 2 (“Effortless self-expression”) was distinguished by moderate identity marking but low defensive and control-oriented behaviors, coupled with the highest need satisfaction. Profile 3 (“Low engagement in self-expression”) showed low scores on all territoriality dimensions and the lowest need satisfaction, indicating a group potentially less connected to their work environment and at greater risk for interpersonal disengagement. The distinct patterns of factor scores for each latent profile are summarized in Table 4, which compares the standardized factor scores across the three identified groups (Fig. 2).

**Table 4:** Comparison of latent profile groups across factor scores

	*N* (%)	BPNs at work		Identity marking		Control marking		Anticipatory defending		Reactionary defending	
		Mean (SD)	95% CI	Mean (SD)	95% CI	Mean (SD)	95% CI	Mean (SD)	95% CI	Mean (SD)	95% CI
Profile 1	155 (59.4%)	0.00 (1.02)	0.16, 0.16	0.12 (0.98)	–0.04, 0.27	0.23 (1.17)	0.05, 0.42	0.36 (1.16)	0.18, 0.54	0.32 (1.20)	0.13, 0.51
Profile 2	79 (30.3%)	0.17 (0.83)	–0.02, 0.35	0.19 (0.90)	–0.01, 0.39	–0.39 (0.57)	− 0.52, − 0.26	–0.56 (0.21)	–0.61, − 0.52	–0.46 (0.09)	− 0.48, − 0.44
Profile 3	27 (10.3%)	–0.48 (1.21)	− 0.96, − 0.01	–1.23 (0.26)	–1.33, − 1.13	–0.19 (0.29)	–0.31, − 0.08	–0.41 (0.12)	–0.46, − 0.36	–0.48 (0.08)	–0.51, − 0.45
ANOVA F		4.38		27.6		11.5		30.3		22.7	
p		0.013		< 0.001		< 0.001		< 0.001		< 0.001	
η²		0.033		0.176		0.082		0.19		0.15	
Significant Post Hoc		2 > 3 (*p* = 0.01)		1 > 3, 2 > 3 (*p* < 0.001)		1 > 2 (*p* < 0.001)		1 > 2, 1 > 3 (*p* < 0.001)		1 > 2, 1 > 3 (*p* < 0.001)	

Note. N (%) = number and percentage of participants in each profile. Means are standardized factor scores (M = 0, SD = 1). 95% CI = 95% confidence interval for the mean

### Demographic and career predictors of latent profile membership

Having established the latent profile structure, we next sought to determine whether key demographic and career-related characteristics could predict membership in these profiles. To this end, we employed a multinomial logistic regression analysis, with age, gender (male, female) years of experience (0–10 years, 11–20 years, more than 21 years) and professional status (non-specialist: intern, resident, specialist candidate; specialist; specialist with multiple board certifications) as predictors and profile membership as the categorical outcome. The overall model demonstrated modest explanatory power (McFadden’s R² = 0.07). Model fit statistics indicated adequate fit for this type of exploratory analysis, with a deviance of 439 and an Akaike Information Criterion (AIC) of 467. Notably, age and gender were included as demographic covariates, but neither showed significant effects on profile membership.

We selected Profile 3 (“Low engagement in self-expression”) as the reference category. Key findings indicate that physicians with more than 21 years of professional experience were over six times more likely to belong to Profile 1 (“Effortful self-expression”) compared to those with 0–10 years of experience (OR = 6.25, *p* = 0.03). We also observed a similar, significant pattern for Profile 2 (Effortless self-expression), with those in the highest experience bracket showing more than five times the likelihood of group membership (OR = 5.53, *p* = 0.047). Regarding professional status, specialists had over four times higher odds of belonging to Profile 1 (“Effortful self-expression”) compared to non-specialists (OR = 4.54, *p* = 0.01). Predictors such as 11–20 years of experience, holding multiple specialist qualifications, age, and gender were not significant.

These results indicate that longer career duration and higher professional status robustly predict a physician’s likelihood of belonging to profiles characterized by heightened territoriality and need satisfaction. In contrast, less experienced and non-specialist physicians have a higher probability of group membership in the “Low Territoriality Disengaged” profile. Detailed results are presented in S2 in Supplementary Materials.

### Workplace well-being in profile groups

Following profile identification and predictor analysis, latent profile groups were compared on occupational well-being outcomes, including work satisfaction, professional fulfillment, interpersonal disengagement, exhaustion, and intention to leave. Statistically significant differences were observed among the latent profile groups for work satisfaction, professional fulfillment and interpersonal disengagement (see Table 5 for group means and confidence intervals), with Profile 3 (“Low engagement in self-expression”) consistently reporting less favorable outcomes across these domains. Specifically, work satisfaction differed significantly among profiles (F(2, 258) = 5.21, *p* = 0.006), and post hoc analyses using the Bonferroni correction revealed that Profile 3 (“Low engagement in self-expression”) reported significantly lower satisfaction compared to Profiles 1 (“Effortful self-expression”) and 2 (“Effortless self-expression”). Professional fulfillment also varied significantly across groups (F(2, 258) = 7.93, *p* < 0.001), with Profile 3 (“Low engagement in self-expression”) again demonstrating significantly lower levels than the other profiles. We found significant differences for interpersonal disengagement (F(2, 258) = 3.65, *p* = 0.027); post hoc comparisons indicated that Profile 2 (“Effortless self-expression”) experienced significantly less interpersonal disengagement than Profile 1 (“Effortful self-expression”). In contrast, no significant differences emerged among the profiles for exhaustion or intention to leave.

In summary, the latent profile analysis revealed three distinct subgroups of physicians based on patterns of basic psychological needs satisfaction and territoriality at work. These profiles exhibited significant differences in work satisfaction, professional fulfillment, and interpersonal disengagement.

**Table 5:** Occupational well-being outcomes by latent profile group

	*N* (%)	Work satisfaction		Professional fulfillment		Interpersonal disengagement		Exhaustion		Intention to leave	
		Mean (SD)	95% CI	Mean (SD)	95% CI	Mean (SD)	95% CI	Mean (SD)	95% CI	Mean (SD)	95% CI
Profile 1	155 (59.4%)	20.6 (6.75)	19.5, 21.7	22.8 (4.73)	22.0, 23.5	15.5 (7.09)	14.3, 16.6	11.7 (4.31)	11.0, 12.4	5.48 (3.43)	4.93, 6.02
Profile 2	79 (30.3%)	20.8 (6.67)	19.4, 22.3	23.5 (4.36)	22.5, 24.5	12.9 (6.03)	11.6, 14.3	10.9 (4.43)	9.87, 11.9	5.38 (3.31)	4.64, 6.12
Profile 3	27 (10.3%)	16.3 (6.67)	13.7, 18.9	19.3 (6.06)	16.9, 21.7	14.6 (7.46)	11.7, 17.6	12.0 (5.54)	9.77, 14.2	6.48 (3.68)	5.02, 7.94
ANOVA F		5.21		7.93		3.65		1.11		1.13	
p		0.006		< 0.001		0.027		0.33		0.324	
η²		0.039		0.058		0.027		—		—	
Significant Post Hoc		1,2 > 3 (*p* = 0.007, 0.008)		1,2 > 3 (*p* = 0.002, < 0.001)		1 > 2 (*p* = 0.022)		—		—	

Note. N (%) = number and percentage of participants in each profile. Mean and standard deviation (SD) are shown for each group; 95% CI = 95% confidence

## Discussion

The present study examined the strategies of niche construction among practicing physicians through basic psychological needs and human territoriality, within the socio-physical context of their workplace. In line with current international trends, we sought to go beyond the phenomenon of burnout and explore the emotional, relational, and environmental strategies that physicians can use to create a personal niche that supports fulfillment. Generally the person-oriented approach aims to discover the configurations of factors that characterize a specific individual’s functioning [52], we emphasize that from an environmental-psychological perspective, the interpretation of strategic behavior is inherently context-dependent. Consequently, such functioning should not be viewed as a stable trait of the individual but rather as an emerging property of the physician–environment transaction.

The study was situated within a socio-ecological framework, wherein social experiences were conceptualized through workplace relationships, particularly along the dimensions of basic psychological needs satisfaction, while the ecological perspective was operationalized via the construction of workplace territoriality. We assumed that processes of personal niche construction would manifest in heterogeneous, individual-level patterns; accordingly, LPA was employed to identify distinct configurations. The study aimed to delineate the latent patterns underlying participants’ organization, to characterize the attributes differentiating each subgroup, and to examine how these distinctive configurations contributed to processes of personal niche construction within the workplace. Although SDT has strong empirical support in workplace research, little effort has been made on how to translate the theory into practical applications [12]. With all these aspects in mind, our results provided valuable insight into the personal niche construction strategies of physicians in healthcare institutions.

### Uniqueness in commonality – characteristics of profiles

The latent profile analyses yielded three distinct profiles that diverged systematically across both territorial strategies, basic psychological needs (Table 4) and occupational well-being outcomes (Table 5). *Profile 1* (“Effortful self-expression”) emerged as the largest subgroup (59.4%), characterized by relatively average levels of basic needs satisfaction, but the highest scores across territoriality dimensions—particularly in control marking (e.g. “Told people the work area is theirs”), anticipatory defending (e.g. “Avoid leaving work area unattended”), and reactionary defending (e.g. “Use locks/passwords so others cannot access their work area”). This suggests that these physicians actively regulate and protect their workspaces as part of their personal niche construction strategy. In terms of outcomes, Profile 1 (“Effortful self-expression”) reported high work satisfaction and professional fulfillment but also demonstrated elevated interpersonal disengagement relative to displayed moderate levels across all territoriality dimensions and moderate level of need satisfaction, suggesting that active assertion and defense of workspace is associated with a sense of autonomy, relatedness, and competence at work. N. B. interpersonal disengagement differs from the depersonalization construct measured by some burnout assessment tools by more specifically assessing empathy and connectedness with others—particularly patients and colleagues [53].

In contrast, *Profile 2* (“Effortless self-expression”) was distinguished by moderate identity marking (e.g. “Decorate the space the way I wanted”) but low defensive (e.g. “Used facial expression to express disagreement towards infringer”) and control-oriented behaviors (e.g. “Told people the work area is theirs”), coupled with the highest need satisfaction. Though Profile 2 (30.3%) displayed the highest satisfaction of basic psychological needs but comparatively low territorial marking and defending behaviors. This pattern may reflect an open, engaged approach to work, where personalization fosters well-being without the need for defensive strategies. In parallel, these physicians reported high work satisfaction and professional fulfillment, coupled with lower levels of interpersonal disengagement than Profile 1 (“Effortful self-expression”). This pattern suggests that Profile 2 represents the most adaptive configuration, where a supportive personal niche fosters need satisfaction and well-being without reliance on strong territoriality. Their strategy seems relational and collaborative, resonating with socio-ecological accounts that emphasize flexible adaptation within supportive environments. Our findings are in line with international research which confirm that moderate and flexible territorial behavior emerges as the most adaptive approach to workspace engagement. Adaptive territorial engagement—characterized by personalization without defensiveness—serves as a socio-ecological mechanism for both individual well-being and collective harmony in shared work environments [54, 55].

*Profile 3 (“Low engagement in self-expression”)* showed low scores on all territoriality dimensions and the lowest need satisfaction, indicating a group potentially less connected to their work environment and at greater risk for interpersonal disengagement. Profile 3 (“Low engagement in self-expression”) (10.3%), though the smallest group, presents the most vulnerable profile. These physicians reported the lowest level of work satisfaction professional fulfillment, exhaustion, intention to leave and a moderate level of interpersonal disengagement compared to Profiles 1 (“Effortful self-expression”) and Profile 2 (“Effortless self-expression”). However, their levels of exhaustion and intention to leave did not significantly differ from the other groups, suggesting that while dissatisfaction is evident, it has not (yet) translated into overt withdrawal intentions. Profile 3 (“Low engagement in self-expression”) thus appears to represent individuals inhabiting a more precarious personal niche, with limited resources for need fulfillment and minimal territorial strategies to secure them.

From a holistic perspective, although the three profiles share several common features, their overall configurations convey distinct meanings. For instance, Profile 1 (“Effortful self-expression”) and Profile 2 (“Effortless self-expression”), are similar in terms of BPNs and identity, yet they differ in how they manage and delineate spatial boundaries. Conversely, Profile 2 (“Effortless self-expression”) and Profile 3 (“Low engagement in self-expression”) show similarities in their low-intensity spatial management but diverge in the expression of BPNs and identity. This pattern suggests that two underlying dimensions may vary across the groups. The first could be described as a “personalization” dimension, reflecting relational experiences and the expression of personal identity through space. The second may be a “boundary regulation” dimension, indicating the extent to which one feels the need to control or protect their territory.

Interpreted along these dimensions: Profile 1 (“Effortful self-expression”) exhibits moderate-to-high personalization and high boundary regulation — indicating personal involvement achieved through territorial control. This pattern may reflect a struggle for personal identity and spatial ownership, with the paradoxical outcome that disengagement is highest here, suggesting ambivalence in their engagement style. Profile 2 represents high personalization and low boundary regulation — implying that strong personal involvement does not require defensive boundary maintenance; the socio-physical space is perceived as secure and psychologically safe. Profile 3 (“Low engagement in self-expression”) shows low personalization and low boundary regulation — indicating minimal personal involvement or investment, an inner disengagement, where strong boundaries are unnecessary because there is little to protect.

### Demographic and career predictors of profile membership

Following the identification of distinct physician profiles based on patterns of territoriality and work engagement, the study examined whether demographic and career-related variables—specifically age, gender, years of experience, and level of qualification (intern, resident etc.)—could predict profile membership. We found that professional experience and level of qualification were significant predictors. Physicians with more than 21 years of experience were more likely to belong to profiles characterized by high territoriality and engagement. Similarly, holding specialist status, as opposed to being a general practitioner, resident, or candidate, significantly increased the likelihood of membership in these profiles. In contrast, age and gender did not emerge as significant predictors. These findings suggest that longer career duration and higher professional standing are associated with more engaged and territorially assertive professional orientations (Profile 1), whereas early-career and lower level of qualification physicians are more likely to occupy disengaged, low-territoriality profiles (Profile 2).

These results provide important insights into how territorial and personal niche construction strategies work in the medical profession. The strong link between professional experience, professional status and membership in profiles that are marked by increased territoriality and engagement indicates that territorial behavior can be a function of accumulated expertise and increased ability to form the professional environment [26, 56]. The more experienced physicians are, the more actively they appear to engage in the construction and defense of personal niches – achieving autonomy, control, and meaning in organizational contexts [57]. This supports the idea that personal niche construction is not a uniform process but varies depending on the position in the professional hierarchy [58].

On the contrary, physicians with less experience and a lower level of qualification are more likely to be occupied with unengaged, low-territorial profiles, which indicates that their ability – or opportunities – are limited to claim control of their work environment. This may reflect structural limitations, such as limited authority and institutional recognition, which limit the ability of early professional to play a proactive role [59]. Overall, these results highlight the strategic role of territoriality in constructing personal niches and allow some physicians to negotiate more favorable working conditions, while others remain restricted or passive [26, 60].

### Well-being outcomes depending on profiles

In the following, we compared the three latent profile groups (Profile 1, Profile 2, Profile 3) on several occupational well-being outcomes such as Work Satisfaction, Professional Fulfillment, Interpersonal Disengagement, Work Exhaustion, Intention to leave. Outcomes were included in the model based on prior research and theoretical relevance. The present findings highlight meaningful distinctions in occupational well-being outcomes across the three latent profiles. Physicians in Profiles 1 (“Effortful self-expression”) and 2 (“Effortless self-expression”), who together represented 90% of the sample, reported significantly higher levels of work satisfaction and professional fulfillment compared to those in Profile 3 (“Low engagement in self-expression”). This indicates that most participants have positive professional performance, whereas smaller subgroups of participating physicians have a marked decrease in satisfaction. However, it is surprising that no significant group differences have occurred in terms of exhaustion or intention to leave, indicating that less satisfaction does not necessarily translate into heightened awareness of turnover or apparent symptoms of stress. A further notable finding was that Profile 1 (“Effortful self-expression”) demonstrated higher levels of interpersonal disengagement than Profile 2 (“Effortless self-expression”), despite comparable levels of satisfaction and fulfillment. This pattern may suggest the presence of compensatory dynamics, whereby physicians maintain positive professional experiences while simultaneously employing interpersonal disengagement strategies to regulate their involvement or preserve personal resources. Such dynamics can be understood within a socio-ecological framework as a form of personal niche adjustment, reflecting how individuals shape and regulate their engagement with the workplace environment to sustain functioning under demanding conditions.

Together, these results highlight the heterogeneity of medical well-being experiences. From a socio-ecological point of view, three latent profiles can be understood as different ways of personal niche construction strategies: Profile 1 (“Effortful self-expression”) reflects a strategy of balancing compliance with partial disengagement as an adaptation preserving resources; Profile 2 (“Effortless self-expression”) suggests a more integrated and sustainable personal niche, characterized by high compliance and low interpersonal disengagement; Profile 3 (“Low engagement in self-expression”) showed a personal niche marked by limited satisfaction and fulfilling opportunities, but without necessarily indicating explicit withdrawal or intention to leave. These models reinforce the idea that occupational well-being is not reduced to linear continuums but consists of various qualitatively different configurations of the individual - environmental relationship [61]. In this sense, the conclusions expand the perspective of personal niche construction, demonstrating that physicians actively create and inhabit different socio-ecological personal niches in the same organizational context and have an impact on how well-being can be supported and sustained.

### The broader context of Hungarian healthcare environments

Our study was conducted among physicians practicing in Hungary thus it could be useful to provide a brief overview of the organizational sociological characteristics of Hungarian healthcare organizations. Healthcare is one of the most bureaucratic organizations and, as such, is characterized by a hierarchy of authority, impersonal interpersonal relationships, expectations of conformity, one-sided communication possibilities, and subordination. Regardless of the specific workplace environment, these structural elements induce numerous factors of anomie in healthcare organizations, such as vulnerability, isolation, and a lack of managerial tolerance and participation [62].

From a context dependent perspective, physicians belonging to Profile 1, who have been working in the system for more than twenty years, have developed their professional independence within an institutionalized framework. They typically enjoy stable status due to their position in the internal hierarchy of the system, but are often unable to gain new professional impetus due to structural rigidity and limited opportunities for innovation [63].

In contrast, Profile 2 represents the transitional, “formative” stage of the medical career, which can be placed around the time of the first board certification. In this phase, physicians are highly mobile, but territorial behaviour is less characteristic of them. Due to the nature of the training system, they must cope with a constantly changing environment (e.g., completing practical training), while at the same time focusing primarily on acquiring knowledge. After obtaining their professional qualifications, members of this group strive to find a workplace where they can fulfill their professional potential, but due to the limited development opportunities in the healthcare system, this “job search” often takes a long time or remains unsuccessful. It is therefore common among young physicians to find that the community they “grew up” in does not welcome them back, while it is not easy to integrate into the higher professional strata. Thus, paradoxically, achieving autonomy goes hand in hand with professional isolation [64].

The Profile 3 appears during the post certificate period, when the physician is no longer a beginner but is not yet a stable member of the system. They constitute the “easily lost” group, as many of them decide at this stage to work abroad (skilled migration), switch to private healthcare, or even leave the medical profession altogether [65]. The Hungarian healthcare system has no means of influencing physicians to stay or enticing them back. It is important to note that migration in itself is not a negative or “harmful” phenomenon. However, if the emigration of physicians from a given country is primarily a “symptom” that reflects the quality of its healthcare system, then a consistent strategy is needed to resolve the situation. It can be assumed that the large-scale exodus of physicians from several countries of East-Central Europe is a “symptom” of a disease, and that migrant physicians would return home after shorter or longer stays, or some of them would not leave at all, if they had confidence in healthcare reforms that were consistent, well-directed, and achievable for them as well [65]. The reasons behind migration decisions may include, among other things, a lack of professional prospects, the quality of life in Hungary, the outlook for Hungarian healthcare, and burnout due to heavy workloads. The emigration balance is skewed toward the 30–49 age group of specialist physicians with Hungarian degrees and greater experience who are leaving their jobs in Hungary [65]. Since these professionals are no longer active members of the system, it is extremely difficult to contact them.

The question may arise as to how the strategies described in Profile 2 lead to Profile 1. In their study examining the resilience of Hungarian physicians, Győrffy and colleagues found that the process is presumably multidimensional [66]. The interaction between the work environment and personality traits can play a decisive role. Personality traits, collegial support, working conditions, and their interactions can have a decisive impact on whether some physicians are able to maintain their mental resilience while others fall victim to burnout [67]. Among the personality traits that predispose individuals to burn out, neuroticism, anxiety, and perfectionism play a prominent role. In contrast, physicians with greater resilience show a higher level of tolerance for uncertainty, are more satisfied with their work, and report stronger collegial support [68]. Overall, it can be said that the operational characteristics detailed above can shape personal niche construction strategies within Hungarian healthcare organizations in a complex way.

### Practical implications of the current study

The results indicate that physician well-being is closely linked to the extent to which work environments support autonomy, professional identity expression, and high-quality interpersonal relations. Consistent with SDT, profiles characterized by higher basic psychological need satisfaction reported greater work satisfaction and professional fulfillment, underscoring the importance of organizational conditions that enable physicians to experience meaningful control and competence in daily practice [11, 69]. In particular, the “Effortless self-expression” profile suggests that workplace designs allowing non-defensive identity marking—such as stable team structures, continuity of clinical roles, and limited opportunities for workspace personalization—may promote well-being without increasing interpersonal disengagement [26, 70].

From an organizational perspective, the overrepresentation of early-career and non-specialist physicians in the Low-Engagement Profile highlights a need for targeted interventions. mentorship programs, professional (or clinician-led) governance, and clearer role articulation may help compensate for limited territorial control and foster psychological need satisfaction among less experienced physicians [71, 72]. Importantly, the absence of profile differences in exhaustion and turnover intention suggests that such interventions may primarily enhance positive dimensions of well-being rather than merely mitigating strain. Overall, the findings argue for moving beyond generic well-being initiatives toward workplace and organizational designs that systematically support autonomy, relatedness, and professional identity as core determinants of physician well-being.

### Limitations

Our research has several limitations. First, although potential profile membership was derived through model classification rather than direct self-reporting, the predictor variables used in the multinomial logistic regression analyses were based on self-reported data collected at a single point in time, which may introduce common method bias. Second, this study employed a cross-sectional design which can show only a snapshot, while personal niche construction is a dynamic process. Additionally cross-sectional data collection does not allow us to infer causal relationships. In the future, it may be worthwhile focusing on the temporal dynamics of using specific places at work and the association with personal niche construction outcomes. Third, the study data were collected through online self-assessment questionnaire, which may be subject to individual subjective biases. Finally, the sample size of the study falls behind the rule of thumb stating that, for an exploratory person-oriented study. a sample size of *N* > 500 may be satisfactory [73]. Therefore, results need to be interpreted with caution, and the correct number of latent profiles must be validated with larger and more diverse samples in future studies. Although stratification by medical specialty would be theoretically informative, it was not feasible due to the limited sample size and high heterogeneity, which would have resulted in very small and statistically underpowered subgroups. To avoid overinterpretation of unstable estimates, we adopted a conservative analytical approach; this limitation is acknowledged, and future studies with larger, specialty-balanced samples are warranted.

### Future research

Long-term and mixed method research is needed to explore the stability of profiles, the potential changes between them, and their impact on the health of physicians over time. Using qualitative methods – such as in-depth interviews - can capture the lived experiences and contextual nuances that shape how individuals construct and maintain their personal niches. These approaches can also help identify emergent strategies and subtle micro-level interactions that quantitative designs might overlook. The extension of methods, including multi-source and behavioral measures, will enhance understanding of the practical implementation of territorial and relational strategies. In addition, identifying antecedents such as personality, leadership and organizational climate can illuminate the conditions under which defense, adaptation, or vulnerable personal niche construction occur. Finally, intervention studies should assess how organizational structures and policies can promote more adaptive personal niches that support both the health and resilience of physicians. The findings should be interpreted primarily within the context of the Hungarian sample and are therefore most directly applicable to the national healthcare setting, subject to the limitations outlined above. Nevertheless, given Hungary’s shared socio-cultural and historical characteristics with other post-socialist and “new democratic” European countries, the observed patterns may be cautiously extendable to similar contexts, although empirical verification is required. More broadly, the results underscore the need for systematic cross-national comparative research in this field. By highlighting context-sensitive configurations of physician well-being and workplace dynamics, the present study aims to serve as a starting point and source of inspiration for future international comparisons that can more precisely delineate the boundary conditions of these findings.

## Conclusion

This study enhances the understanding of physicians’ well-being by integrating socio-ecological and model-based approaches to explore how individuals actively construct personal niches. A joint study of basic psychological needs and territorial behaviors revealed three different configurations, reflecting the heterogeneous strategies of personal niche construction. The findings show that workplace well-being is not a static feature, but a dynamic adaptation process in which autonomy, competence and relatedness are maintained by regulation of personal and environmental limits. Experienced, multi-specialist physicians were more likely to occupy personal niches characterized by active engagement and territorial assertion, while early-career professionals tended to exhibit low territoriality and interpersonal disengagement, underscoring structural constraints in workplace agencies. Profiles also differed significantly in professional fulfillment, satisfaction, and interpersonal disengagement, indicating that satisfaction and territorial strategies jointly shape well-being outcomes. In general, these results highlight the relevance of socio-ecological perspectives in occupational health, demonstrating that physicians’ capacity to construct, claim, and maintain their personal niches serves as a central mechanism of resilience in complex healthcare environments. Promoting conditions that enable autonomy, support, and balanced spatial ownership may thus foster sustainable well-being and engagement across professional trajectories.

## Supplementary Information

Below is the link to the electronic supplementary material.

Supplementary Material 1

## Data Availability

The datasets used and/or analyzed during the current study are available from the corresponding author on reasonable request.

## Abbreviations

AIC: Akaike Information Criterion
BIC: Bayesian Information Criterion
BLRT: Bootstrap Likelihood Ratio Test
BPNs: Basic Psychological Needs
COVID-19: Coronavirus Disease 2019
EFA: Exploratory Factor Analysis
LPA: Latent Profile Analysis
P–E fit: Person-Environment fit
SPFI: Stanford Professional Fulfillment Index
SPFI-H: Stanford Professional Fulfillment Index - Hungarian
WAMI: Work and Meaning Inventory
SDT: Self-Determination Theory
SRH: Subjective Rated Health
SWWS: Satisfaction with Work Scale
