# Supplementary material for: Fitting in, Standing out: Latent profiles of personal niche construction strategies at the workplace among Hungarian physicians
Source: BMC Health Serv Res. 2026 Mar 10;26:540. doi: 10.1186/s12913-026-14317-4 (PMC13088593; doi:10.1186/s12913-026-14317-4)
Supplement: Supplementary file 1 — Supplementary Material 1 [file 12913_2026_14317_MOESM1_ESM.docx]

**Table S1:** Fit indices of the LPA models

|  | **Model 1** | | | | **Model 2** | | | | **Model 3** | | | | **Model 4** | | | |
| --- | --- | --- | --- | --- | --- | --- | --- | --- | --- | --- | --- | --- | --- | --- | --- | --- |
| k | AIC | BIC | Entropy | BLRT / p | AIC | BIC | Entropy | BLRT / p | AIC | BIC | Entropy | BLRT / p | AIC | BIC | Entropy | BLRT / p |
| 2 | 3731 | 3788 | 0,05 | -0,2 / 1 | 3180 | 3255 | 0,90 | 560,6 / < .01 | 3682 | 3774 | 0,87 | 68,6 / < .01 | 2957 | 3104 | 0,96 | 822,9 / < .01 |
| 3 | 3669 | 3748 | 0,76 | 73,1 / < .01 | 3097 | 3211 | 0,90 | 104,67 / < .01 | 3596 | 3710 | 0,83 | 98 / < .01 | 2818 | 3039 | 0,97 | 181,4 / < .01 |
| 4 | 3531 | 3631 | 0,81 | 150,6 / < .01 | 3097 | 3250 | 0,90 | 22 / 0,34 | 3559 | 3694 | 0,90 | 49,3 / < .01 | 2802 | 3098 | 0,96 | 57,6 / 0,36 |
| 5 | 3599 | 3720 | 0,65 | -56,1 / 0,48 | 3034 | 3226 | 0,84 | 85,4 / < .01 | 3539 | 3696 | 0,84 | 31,1 / < .01 | 2711 | 3082 | 0,93 | 132,9 / < .01 |
| 6 | 3609 | 3751 | 0,65 | 2,3 / 0,96 | 3008 | 3240 | 0,85 | 47,5 / < .01 | 3551 | 3730 | 0,71 | 0,09 / 0,98 | 2720 | 3166 | 0,94 | 33,1 / 0,81 |
| 7 | 3539 | 3703 | 0,68 | 81,5 / 0,< .01 | 2977 | 3248 | 0,86 | 52,7 / < .01 | 3522 | 3722 | 0,66 | 41,18 / < .01 | 2656 | 3176 | 0,94 | 106,3 / < .01 |
| 8 | 3550 | 3735 | 0,65 | 1,6 / 0,97 | 2963 | 3274 | 0,86 | 35,9 / < .01 | 3534 | 3755 | 0,63 | 0,24 / 0,99 | 2748 | 3343 | 0,94 | -49,4 / 1 |

*Note*: bold values represent local minima for AIC and BIC and the first non-significant BLRT value

Model 1: equal variances and zero covariances; Model 2: varying variances and zero covariances; Model 3: equal variances and covariances; Model 4: varying variances and covariances

**Table S2**: Multinomial Logistic Regression of Latent Profile Group Membership Based on Career-Related Characteristics Among Physicians (Profiles Defined by Basic Psychological Need Satisfaction and Workplace Territoriality)

|  | Latent Profile Membership (reference group: Profile 3, *n* = 27 | | | | | |
| --- | --- | --- | --- | --- | --- | --- |
|  | **Profile 1** | | | **Profile 2** | | |
|  | OR | 95% CI | *p* | OR | 95% CI | *p* |
| **Professional experience** |  |  |  |  |  |  |
| 11 - 20 years (vs 0 - 10 years) | 0.66 | 0.24, 2.14 | 0.49 | 0.95 | 0.27, 3.32 | 0.94 |
| 21 years+ (vs 0 - 10 years) | 5.22 | 1.11, 24.52 | 0.04 | 4.97 | 0.99, 25.04 | 0.05 |
| **Professional status** |  |  |  |  |  |  |
| Specialist (vs non-specialist) | 4.24 | 1.33, 13.51 | 0.02 | 2.81 | 0.81, 9.72 | 0.10 |
| Specialist with multiple board certifications (vs non-specialist) | 3.09 | 0.66, 14.42 | 0.15 | 2.69 | 0.53, 13.51 | 0.23 |
